# Inhibition of HBV Replication by a Fully Humanized Neutralizing Antibody In Vivo and In Vitro

**DOI:** 10.32607/actanaturae.27457

**Published:** 2025

**Authors:** Zhipeng Zhang, Yanqin Ma, Yan He, Dong Wang, Huaien Song, Kun Yue, Xiaomei Zhang

**Affiliations:** Laboratory of Pharmaceutical Engineering, School of Life Science and Health Engineering, Jiangnan University, Wuxi, 214122 China; Suzhou Hepa Thera Biopharmaceutical Co., Ltd., Shanghai, 200120 China

**Keywords:** Neutralizing antibody, CDC effect, HBsAg

## Abstract

Neutralizing antibodies are capable of specifically binding to the HBsAg virus, thereby preventing HBV infection and subsequently reducing viral antigen load in both the liver and systemic circulation. This has significant implications for restoring the postnatal immune function. By utilizing the phage antibody library technology, we successfully screened a fully humanized neutralizing antibody targeting the hepatitis B surface antigen. The antiviral activity was assessed in primary human hepatocytes (PHHs) by determining the EC_50_ values for HBeAg and HBsAg biomarkers in HBV types B, C, and D; no cytotoxicity was observed within the tested concentration range. Furthermore, HT-102 exhibited no ADCC effect but displayed a weak CDC effect along with a dose-dependent response. We established an AAV/HBV mouse model and observed significant dose-dependent reduction in HBsAg and HBV DNA levels for both the medium-dose and highdose groups. The immunohistochemical staining data showed dose-dependent reduction in HBsAg expression in the liver, with high-dose group exhibiting minimal positive expression. Finally, a mild immune response was induced, while reducing the burden of antigen–antibody complexes circulating within the system. Consequently, strain on the patient’s immune system was alleviated by effectively slowing down CD8^+^T lymphocyte depletion, and functional cure was ultimately achieved as intended.

## INTRODUCTION

Hepatitis B virus (HBV) infection is a common public health problem worldwide; 5–10% of persistent HBV infections following acute hepatitis B develop into chronic liver disease, including chronic active hepatitis, cirrhosis, and primary liver cell carcinoma [1]. Although nucleic acid analogs effectively prevent the risk of HBV reactivation and completely eliminate the possibility of hepatitis outbreak, the probability of functional cure is extremely low, and it still causes serious damage to the liver and even the occurrence of liver cancer [2].

Currently, prevention of hepatitis B virus infection primarily involves active and passive immunization [3]. Active immunization entails administering the hepatitis B vaccine, making it one of effective measures for preventing hepatitis B transmission [4]. Passive immunization involves administering hepatitis B immune globulin (HBIG), which is mainly used to prevent mother-to-child transmission (in combination with the hepatitis B vaccine) [5]. Research has demonstrated that a combination of both HBIG and the hepatitis B vaccine is more effective in reducing the chronic infection rate [6]. Most HBIG is derived from positive serum containing anti-HBsAg, which limits its large-scale production and poses a risk for blood-borne infectious diseases because it is originating from serum sources. Despite the transition from blood-derived vaccines to genetically engineered ones, there is an urgent need to develop genetically engineered antibodies against anti-HBs as a replacement for HBIG [7]. The phage antibody library technology offers an alternative solution to address this issue.

The present study mainly introduced a new fully humanized neutralizing antibody (HT-102), which was in phase 1 clinical stage (Chinese Clinical Trial Registry No. ChiCTR2200072837). The phage display Fab libraries were constructed using the established methods [8, 9] based on targeted genes isolated from PBMCs of 18 donors who had received hepatitis B virus vaccination. Total cellular mRNAs were extracted using an RNeasy Mini Kit (Qiagen), and cDNA synthesis was primed with oligo (dT) using a Transcriptor High Fidelity cDNA Synthesis Kit (Roche). The light and heavy chain genes were amplified from the cDNA by PCR and sequentially cloned into the pComb 3H vector using a standard protocol [10]. Fab antibody preparations were tested and screened by indirect ELISA using 96-well plates coated with 0.5–1 μg of purified S protein, with horseradish peroxidase (HRP)-conjugated anti-human Fab used as a secondary antibody. Following the evaluation of the clones, HT-102 was selected as the final monoclonal antibody due to its superior performance in terms of anti-HBsAg titer, Fab expression levels, and binding affinity [11]. The primary mechanism involves specific binding to the S antigen on the surface of the HBV virus [12], which prevents its interaction with cell receptors and subsequent entry into cells, consequently impeding HBV infection in uninfected cells [13].

## MATERIALS AND METHODS

***In vitro***

The following commercial cell lines were used for *in vitro *efficacy assays: PHHs (Wuxi Apptec, cat. # LGI, China), Myrcludex B (Wuxi AppTec, cat. # P1214012, China), Cell PBMC (HemaCare, cat. # 20063062, USA), HepG2-HBsAg and Raji cells (Wuxi AppTec, China). Detailed information regarding the HBV virus is provided in *Table 1 *(see Appendix). The following commercial test kits were utilized in this experiment: LDH assay kit (Promega, cat. # G1780, USA), CCK-8 (Li Ji Biochemicals, cat. # AC11L057, China), HBsAg ELISA kit (Autobio Inc., cat. # CL-0310, China), and HBeAg ELISA kit (Autobio Inc., cat. # CL-0312, China). The main instruments used in this experiment include an enzyme-linked immunosorbent assay (ELISA) reader (Molecular Devices, USA), a centrifuge (Beckman Coulter, USA), and a cell counter (Countstar, China).

*Anti-HBV efficacy. *On day 0, PHH cells were recovered and adjusted to a suitable density of 1.32 × 10^5^ cells/well before being seeded into 48-well cell plates at a concentration of 20 µg/ml. On day 1, HT-102 was prepared at starting concentrations of 20, 5, 1.250, 0.313, 0.078, 0.020, and 0.005 µg/ml to be mixed with type B, type C, and type D HBV viruses for 1 h before being added to the cells. Similarly, Myrcludex B was prepared at starting concentrations of 100, 25, 6.250, 1.563, 0.391, 0.098, and 0.024 nM. On day 8, the cell culture supernatants were collected for CCK-8 assay to determine cell viability as well as ELISA analysis for HBeAg and HBsAg detection. The HBsAg inhibition rate (%) and HBeAg inhibition rate (%) were calculated as (1 – [HBsAg or HBeAg test sample concentration / HBsAg or HBeAg medium control concentration]) × 100%, respectively. Cell viability% was determined as (test sample absorbance – blank average absorbance)/ (medium control average absorbance – blank average absorbance) × 100%. The data were analyzed using the log(inhibitor) vs response-variable slope method in the GraphPad Prism software to obtain the EC_50_ and CC50 values of the compound against HBV.

*The antibody-dependent cellular cytotoxicity (ADCC) and complement-dependent cytotoxicity (CDC) effects.* The binding rate of the tested antibody to the target cell was verified as follows. Different concentrations of HT-102 (0.1, 1, 10, and 100 µg/ml) were prepared and incubated with HepG2-HBsAg stably transfected cells at 4°C for a specified duration. A negative control was included simultaneously. Fluorescent secondary antibody APC-anti-human IgG Fc (Jackson ImmunoResearch, cat. # 109-605-098, USA) was added and incubated. Finally, flow cytometry was employed to determine the binding rate.

**ADCC: **On day 1, PBMC cells were adjusted to a density of 2 × 10^6^ cells/ml. Raji and HepG2-HBsAg stable transfection cell lines were also adjusted to a density of 4 × 10^5^ cells/ml. The antibodies, including positive control Rituximab (MedChemExpress, cat. # HY-P9913, USA) and negative control IgG1 (Genenode, cat. # 91001B, China), were then prepared at concentrations ranging from 100 to 0 μg/ml. The LDH test was performed in strict accordance with the manufacturer’s instructions provided in the LDH assay kit. Killing rate = (Test sample absorbance – Low control absorbance – PBMC absorbance) / (High control absorbance – Low control absorbance) × 100%. ADCC% = (killing rate of test sample –killing rate of no-antibody control) × 100%.

**CDC: **The cell density of Raji and HepG2 cells was separately adjusted to 4 × 10^5^ cells/ml. HT-102 antibody, positive control Rituximab, and negative control IgG1 were prepared at concentrations ranging from 100 to 0 μg/ml. Next, the lysis solution was introduced into each well for lysing the cells thereby releasing LDH (lactate dehydrogenase). The instructions provided in the LDH kit were followed meticulously to conduct the LDH test. Complement-Mediated Cytotoxicity of Target Cells: Killing rate = (Test sample absorbance – Low control absorbance) / (High control absorbance – Low control absorbance) × 100%. CDC% = (killing rate of test sample –killing rate of no-antibody control) × 100%.

***In vivo***

The recombinant rAAV8-1.3HBV (type D, ayw; batch number: awy1-P4-220301) was procured from Shanghai Wuxi AppTec. The primary reagents and instruments used are detailed in *Table 2* and *Table 3* (see Appendix).

**Fig. 1:**
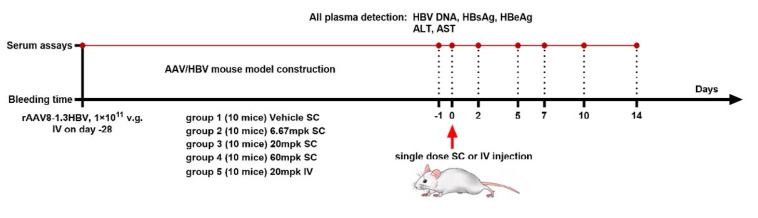
Experimental design

*Evaluation of the anti-HBV activity by a single dose injection. *Before injection, rAAV8-1.3HBV was prepared in sterile PBS at a concentration of 1 × 10^11^ v.g./200 µl. Injections were administered to 60 mice via the tail vein. After screening, 50 mice were divided into five groups and designated as group 1 through group 5. Blood plasma was collected before detecting HBV DNA, HBsAg, and HBeAg on days 14 and 21 after virus injection. On day 0 (28 days after virus injection), four groups of mice were subcutaneously injected with a blank vehicle or a test compound solution, while the fifth group of mice was injected with the test compound solution via the tail vein. Blood plasma samples were collected from all mice via the submandibular vein on days -1, 2, 5, 7, 10, and 14, and used to detect HBV DNA, HBsAg, and HBeAg. These blood plasma samples were also used to detect ALT and AST on days -1, 7, and 14 (Appendix, *Fig. S1*). The experimental protocol is shown in *Fig. 1*. Data are presented as mean ± standard deviation of each group of mouse samples, unless otherwise specified.

**Fig. 2:**
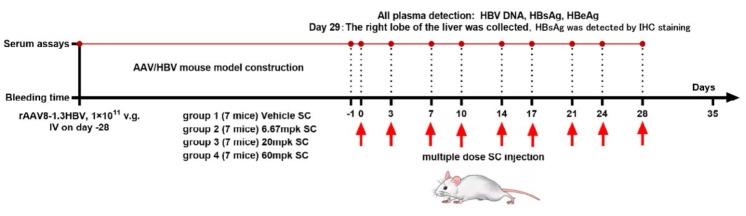
Experimental design

*Evaluation of the anti-HBV activity by multiple dose injection. *All 35 mice successfully received 200 µl of the rAAV8-1.3HBV solution via the tail vein. After screening, 28 mice were selected into groups and labeled as group 1 through group 4. Blood samples were collected from infected mice via the subclavian vein on days 24 and 44 post-infection and stored at –80°C for detecting HBV DNA, HBsAg, and HBeAg [14]. On day 0, mice in groups 1– received subcutaneous injections of either a vehicle or a test compound. Blood samples were collected from all mice through the subclavian vein on days -1, 1, 5, 8, 12, 15, 19, 22, 26, and 29 post-infection for detecting HBV DNA, HBsAg, and HBeAg. All mice were sacrificed by CO_2_ inhalation on day 29, and the right lobe of the liver was harvested and preserved in formaldehyde, transferred to PBS, and embedded into paraffin blocks to conduct IHC staining for detecting HBsAg. *Figure 2* illustrates the experimental protocol design. The results of HBV DNA, HBsAg, HBeAg analysis are presented as the mean value ± standard deviation per group of mouse samples, unless otherwise specified.

## RESULTS

***In vitro *anti-HBV efficacy**

**Fig. 3:**
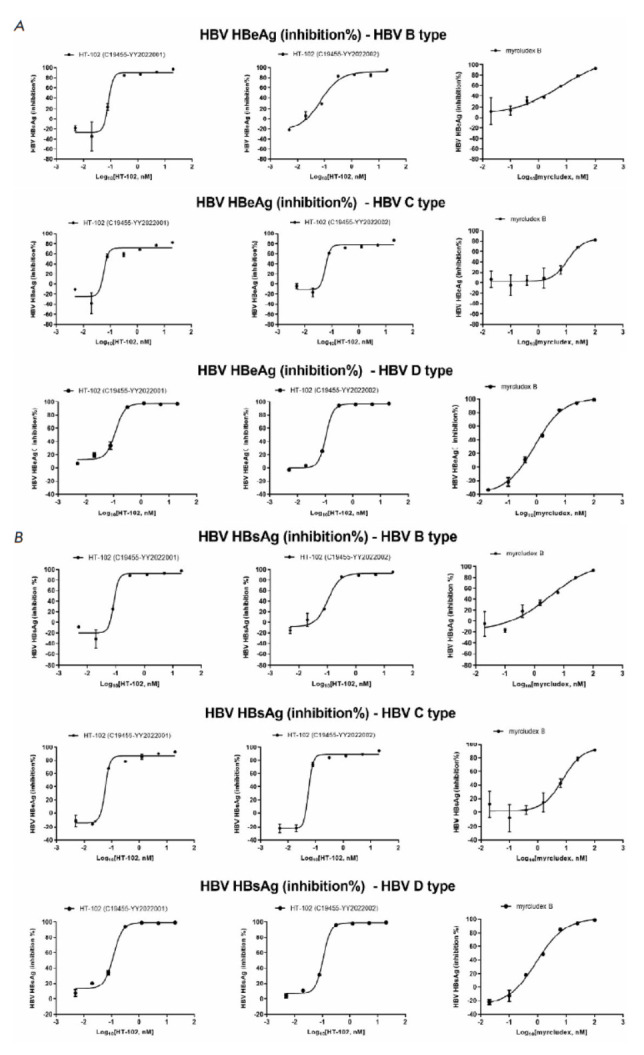
(A) The fit curve for the inhibition of HBeAg by neutralizing antibody. (B) The fitting curve for the inhibition of HBsAg by neutralizing antibody targeting HBV surface antigens. Error bars represent standard errors

The experimental protocol was designed to validate the *in vitro *antiviral activity within the PHHs system. Myrcludex B exhibited expected inhibition against HBeAg subtypes B, C, and D with EC_50_ values of 8.583, 11.180, and 0.853 nM, respectively, as well as against HBsAg subtypes B, C, and D with EC_50_ values of 3.358, 7.545, and 0.908 nM, respectively [15, 16]. HT-102 (batch number: C19455-YY2022001(C)) demonstrated EC_50_ values of 0.083, 0.057, and 0.117 µg/ml for inhibition of HBeAg subtypes B, C and D, and EC_50_ values of 0.084, 0.058, and 0.119 µg/ml for inhibition of HBsAg subtypes B, C, and D. HT-102 (batch number: C19455-YY2022002) showed EC_50_ values of 0.072, 0.058, and 0.107 µg/ml for inhibition of HBeAg subtypes B, C, and D, and EC_50_ values of 0.104, 0.055, and 0.108 µg/ml for inhibition of HBsAg subtypes B, C, and D. The fit curves are shown in *Fig. 3*.

**Fig. 4:**
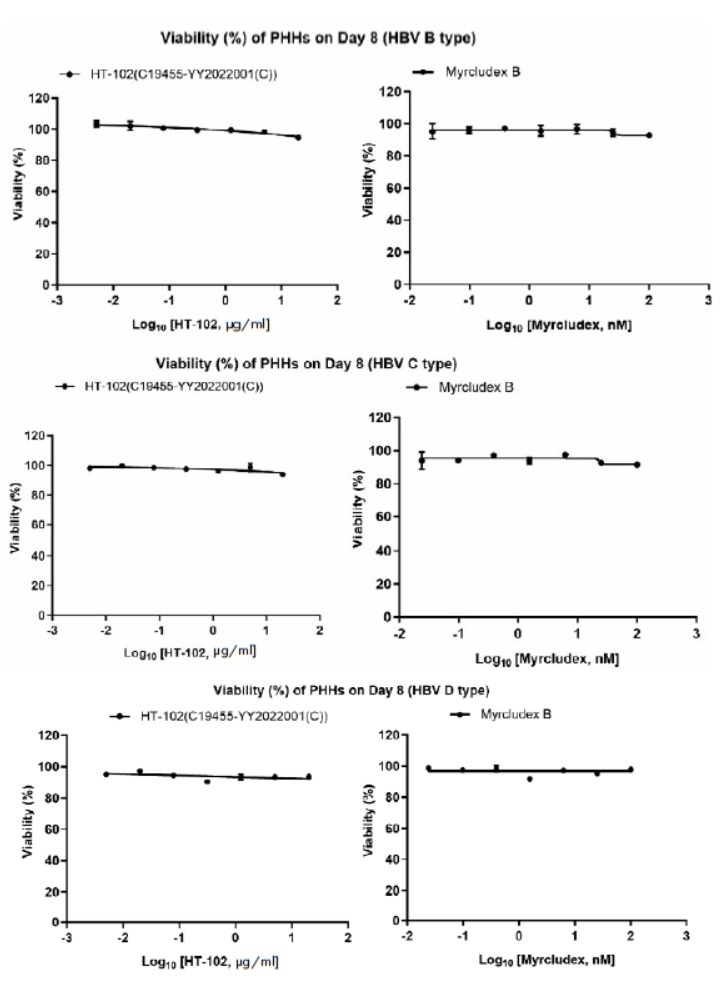
The fit curves for cell viability. Error bars represent standard errors

A microscopy study revealed that neither HT-102 (batch number: C19455-YY2022001(C)) nor myrcludex B exhibited an apparent toxicity against PHHs cells. This finding was further supported by the results obtained from CCK-8 detection. *Figure 4* shows the cell viability curve.

**The antibody-dependent cellular cytotoxicity (ADCC) and complement-dependent cytotoxicity (CDC) effects**

**Fig. 5:**
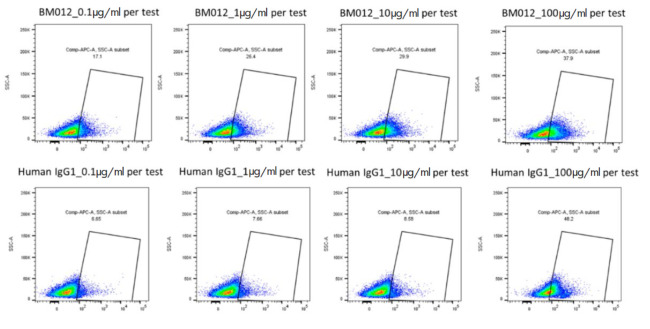
The efficacy of the neutralizing antibody and negative control antibody in their binding to the target cells

HepG2 cells stably expressing HBsAg protein were used as target cells. When evaluating the binding efficiency of the neutralizing antibody to these target cells [17], flow cytometry results demonstrated a concentration- dependent increase in binding rates between the target cells and various concentrations (0.1, 1, 10, and 100 µg/ml) of HT-102(BM012). The highest binding rate, 37.9% at a concentration of 100 µg/ml, was observed with HepG2-HBsAg stably transfected cells. In contrast, the binding rates of the negative control antibody were significantly lower than those of HT-102(BM012) at the same concentrations. However, it is worth noting that at a concentration of 100 µg/ml, the binding rate was elevated (48.2%) for the negative control antibody, suggesting potential non-specific staining due to excessive concentration. These findings shown in *Fig. 5*.

Evaluation of the ADCC activity revealed that Rituximab exhibited a significant dose-dependent ADCC activity within its specified range (13.57– 53.03%) [18, 19]. The negative control antibody, human IgG1, exhibited an ADCC activity of -7.35%. Concentrations of the positive and negative controls used in the test are listed in *Table 4 *(see Appendix). The test antibody HT-102(BM012) displayed no detectable ADCC activity within its specified range (0.0064–100 μg/ml) (*Table 5*, see Appendix).

During further assessment of the CDC effect of the test antibody, it was observed that Rituximab exhibited a CDC effect ranging from 0.68 to 15.59% within its tested concentration range (0.0064–100 µg/ml). The HT-102 (BM012) showed a CDC effect ranging from -0.71 to 5.23% within its tested concentration range (0.0064–00 µg/ml), while the negative control human IgG antibody had a CDC effect value of -0.13%. These findings indicated that HT-102 (BM012) exhibited a weak but dose-dependent CDC effect. The detailed results are available in *Table 6* and *Table 7* (see Appendix).

**Evaluation of the *in vivo *anti-HBV activity by a single dose injection**

**Fig. 6:**
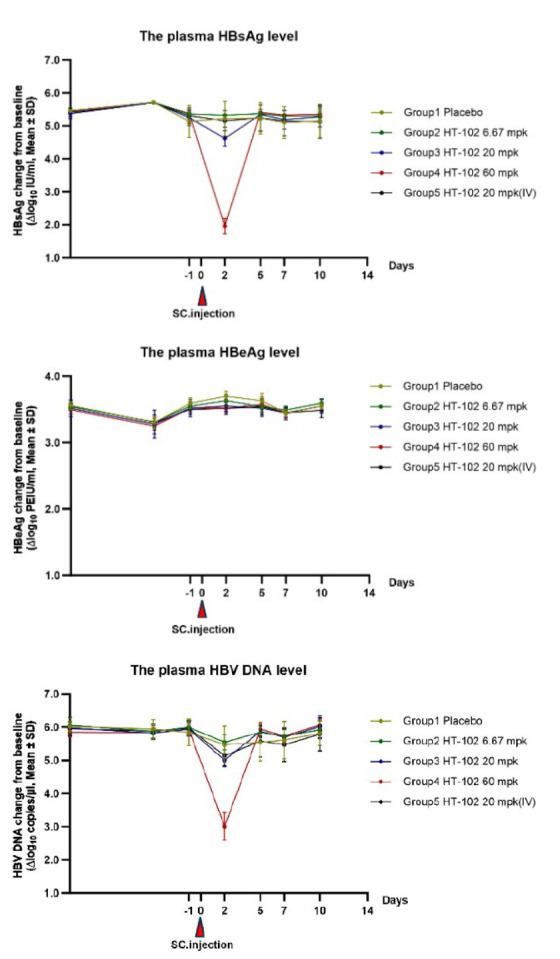
The effects of the test compound on HBsAg, HBeAg, and HBV DNA in the plasma of AAV/HBV mice. The plasma levels of HBsAg and HBeAg in mice were quantified by ELISA, while the HBV DNA level was determined by quantitative PCR analysis. Error bars represent standard errors

The levels of HBeAg, HBsAg, and HBV DNA in mice in the vehicle group remained relatively stable throughout the experiment, fluctuating within the ranges of 3.30–3.70 log10 PEIU/ml for HBeAg, 5.10–5.72 log10 IU/ml for HBsAg, and 5.47–6.02 log10 copy/μL for HBV DNA during the experimental period [20]. Low-dose group (6.67 mpk, SC): on day 0, mice in group 2 were compared to the vehicle group. No significant reduction was observed in plasma levels of HBeAg, HBsAg, and HBV DNA. Medium-dose group (20 mpk, SC): mice in group 3 were compared to the vehicle group; on day 2, a slight decrease was observed in plasma levels of HBeAg (-0.15 log10 PEIU/ml; *p* < 0.01), HBsAg (-0.60 log10 IU/ml; *p* < 0.01), and HBV DNA (-0.47 log10 copy/μL; *p* < 0.05), but these levels rebounded by day 10 after treatment. High-dose group (60mpk, SC): mice in group 4 were compared to those in the vehicle group; on day 2, there was a slight decline in plasma level of HBeAg (-0.18 log10 PEIU/ml; *p* < 0.01), and significant decrease in both HBsAg (-3.26 log10 IU/ml; *p* < 0.01) and HBV DNA levels (-2.47 log10 copy/μL;*p* < 0.01). However, identically to the observations in the medium-dose group, the levels of HBeAg, HBsAg, and HBV DNA returned to the baseline. In the medium- dose group (20 mg/kg, IV), mice in group 5 were injected via the tail vein. Compared to the vehicle group, there was slight reduction in plasma HBeAg and HBV DNA levels on day 2 (0.12 log10 PEIU/ml (*p* < 0.01) and 0.41 log10 copies/μL (*p* < 0.01), respectively). However, by day 10 post-dose, the HBsAg levels returned to the level of the vehicle group. The results of the entire experiment are presented in *Fig. 6*.

**Evaluation of the *in vivo *anti-HBV activity by multiple dose injection**

**Fig. 7:**
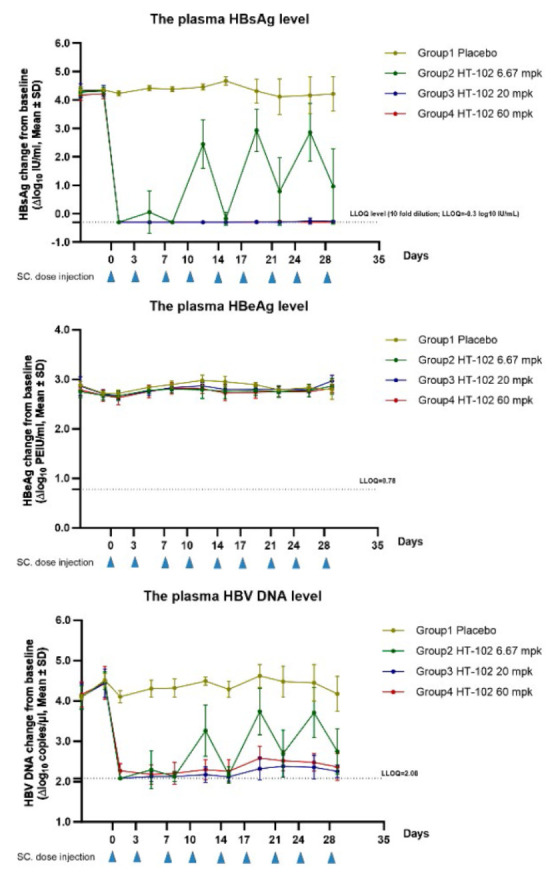
The effects of the test compound on plasma levels of HBsAg, HBeAg, and HBV DNA in AAV/HBV mice. The plasma levels of HBsAg and HBeAg in mice were quantified by ELISA, while the HBV DNA level was determined by quantitative PCR analysis. Error bars represent standard errors

Group 2 (6.67 mg/kg, SC): HT-102 was administered subcutaneously at a dose of 6.67 mg/kg every three days. Compared to the vehicle group, the plasma HBeAg level in mice slightly decreased from day 8 to day 19 post-dose; the mean decrease ranged from 0.09 to 0.19 log10 PEIU/ml (*p* < 0.05). The other time points were similar to those in the vehicle group. The plasma HBsAg level in mice was significantly reduced on day 1 after the first administration and decreased to the lower limit of quantification (LLOQ); the plasma level of HBsAg fluctuated between day 5 and day 29. A significant decline was observed on days 8, 15, 22, and 29; the mean decrease was 4.67, 4.84, 3.33, and 3.26 log10 IU/ml (*p* < 0.01), respectively. Compared with the vehicle group, the plasma level of HBV DNA in mice was significantly lower after the first administration of HT-102; subsequently, on days 5 through 29, there were fluctuations in the plasma levels of HBV DNA related to the administration time, with a significant decrease observed on days 8, 15, 22, and 29 (the mean decrease being 2.20, 2.12, 1.78, and 1.43 log10 copies/μL (*p* < 0.01), respectively). Plasma levels of HBeAg in group 3 mice were slightly decreased (20 mg/kg) compared to the vehicle group on days 5 and day12 through day 19 post-dose. The plasma levels of HBsAg in mice were significantly reduced on days 1 through 29, reaching the LLOQ value. The mean decrease in the HBsAg level was between -4.42 and -4.97 log10 IU/ml (*p* < 0.01). In a similar manner, the plasma levels of HBV DNA in mice were significantly decreased at all time points between day 1 and day 29 compared to those in the vehicle group and slightly reduced, approaching the LLOQ value. The mean decrease in HBV DNA was between -1.92 and -2.32 log10 copy/μl (*p* < 0.01). In group 4 mice, the serum levels of HBeAg were slightly decreased on days 12 through 19 compared to those in the vehicle group; the mean reduction range was -0.15 to -0.23 log10 PEIU/ml (*p* < 0.05), while results similar to those in the vehicle group were observed for other time points. Furthermore, the serum HBsAg levels were significantly reduced on days 1 through 29, reaching the LLOQ value, the mean reduction range being -4.40 to -4.97 log10 IU/ml (*p* < 0.01). In a similar manner, the serum levels of HBV DNA significantly decreased from day 1 to 29 and approached the LLOQ value, with mean reduction range of -1.81 to -2.20 log10 copy/μl (*p* < 0.01). Detailed graphs are shown in *Fig. 7*.

**Fig. 8:**
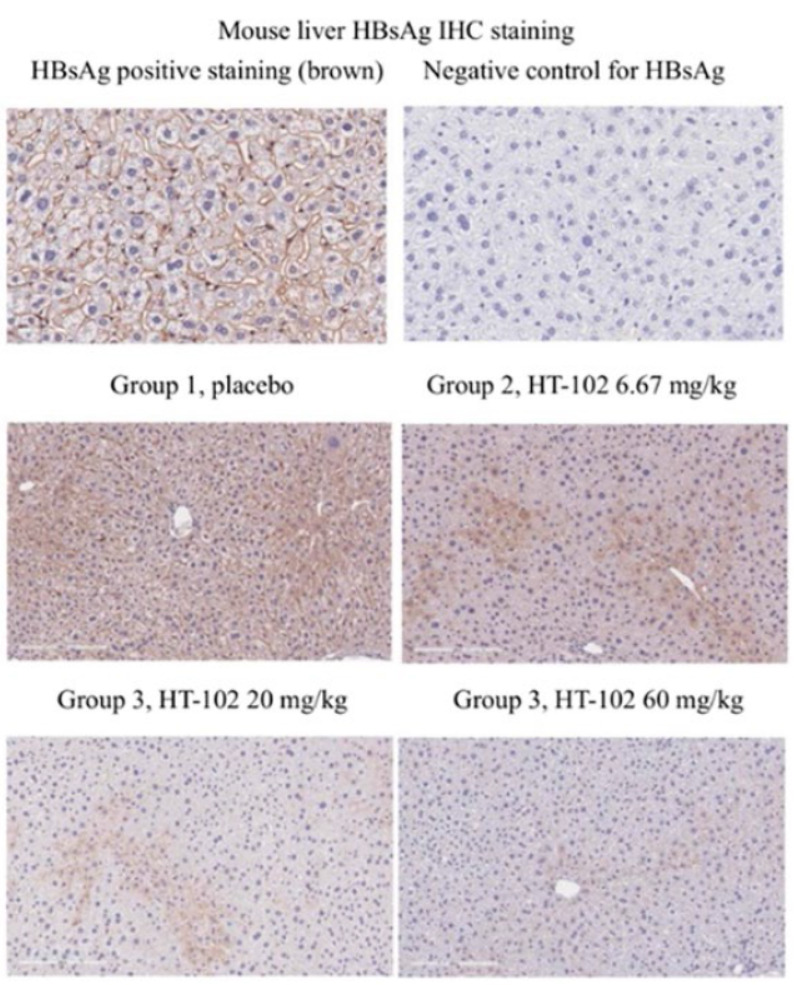
Immunohistochemical staining of HBsAg was performed in mouse liver samples to evaluate the anti- HBV activity in the AAV/HBV mouse model through multiple dose injections. The HBsAg levels in the mouse liver were determined by IHC staining, compared with positive and negative controls; groups 1 to 3 were sampled for liver tissue staining on day 29

*Figure 8* shows HBsAg expression in the liver for each mouse group. All the liver tissue sections harvested from AAV/HBV-infected mice were characterized by specific localization of HBsAg. Moreover, equine anti-HBsAg polyclonal antibody was used to stain brown for the positive control in IHC staining. Positive HBsAg expression was predominantly concentrated within the hepatic sinusoidal region and exhibited a radial distribution [21, 22]. Microscopic examination revealed a significant dose-dependent reduction in HBsAg expression in liver tissue samples from the low-dose, medium-dose, and high-dose groups compared to the placebo group. Notably, the lowest level of HBsAg positive expression was observed for mice in the high-dose group.

## DISCUSSION

The excessive release of HBsAg in chronic HBV patients leads to tolerance to antibodies and cell-mediated immune responses, which currently is a major obstacle to eradication of the virus [23, 24]. Therefore, it is crucial to identify approaches that can overcome immune tolerance and enable hosts to generate effective immune responses capable of clearing the virus and preventing further HBV infection [25, 26].

We conducted *in vitro *assays to evaluate the antiviral activity of the compound against hepatitis B virus (HBV) types B, C, and D. The HBeAg and HBsAg levels were quantified by ELISA, while human primary hepatocytes (PHHs) were employed for assessing the efficacy of the compound. Furthermore, no cytotoxic effects were observed within the tested concentration range. This study revealed no ADCC effect; however, HT-102 exhibited a weak and dose-dependent CDC effect. Subcutaneous administration of the test antibody at medium and high doses effectively reduced the HBeAg, HBsAg, and HBV DNA levels, being indicative of a significant dose-dependent response. Analysis of the ALT and AST levels in blood samples revealed no significant elevation in the mean postdose levels among the treatment groups, indicating that there was no adverse impact on liver function. Furthermore, repeated subcutaneous low-dose, medium- dose, and high-dose injections effectively reduced the HBeAg, HBsAg, and HBV DNA levels, while exhibiting a favorable dose-dependent effect across all dosage groups. The immunohistochemical staining data revealed significant decline in HBsAg expression in the liver tissue samples; mice in the high-dose group exhibited the lowest HBsAg positive expression.

The results of both *in vivo *and *in vitro *pharmacological experiments indicate that the *in vivo *studies yielded some unexpected outcomes. Specifically, single medium- and high-dose administration led to a rapid rebound in HBsAg levels. After multiple low-dose administrations, HBsAg biomarkers exhibited cross-correlation between rebound and inhibition. However, after administration of multiple medium and high doses, HBsAg biomarkers remained at or below the lower limit of detection. The low-dose group exhibited unsatisfactory findings, two fundamental reasons underlying this observation. First, immunogenicity played a crucial role. Although neutralizing antibodies had shown promising clinical effects, fully humanized antibodies may elicit immune responses in mice, resulting in production of antidrug antibodies (ADAs). ADAs could neutralize activity of the antibody drug, affect drug clearance and bioavailability, alter the pharmacokinetic characteristics of drugs, as well as interfere with or impede therapeutic efficacy [27, 28, 29]. A fluctuating rebound effect was observed in the medium- dose group. It was possible to detect the presence of antidrug antibody (ADA) in the blood serum of mice and assess changes in its pharmacokinetic properties, as well as conduct research on constructing a humanized liver chimeric mouse model infected with HBV. Second, the initial administration of neutralizing antibodies may induce a negative feedback regulation, thereby further stimulating the release of viral particles from infected hepatocytes, leading to the inefficacy observed in the low-dose group, while the medium-dose group exhibited a fluctuating rebound in the mouse model of HBV infection. However, the high-dose group directly neutralized both extracellular circulating HBV viral particles and newly secreted ones from infected hepatocytes, consistently maintaining them below the limit of quantification (LLOQ). This finding provided valuable insights for subsequent clinical dosing regimens [30].
